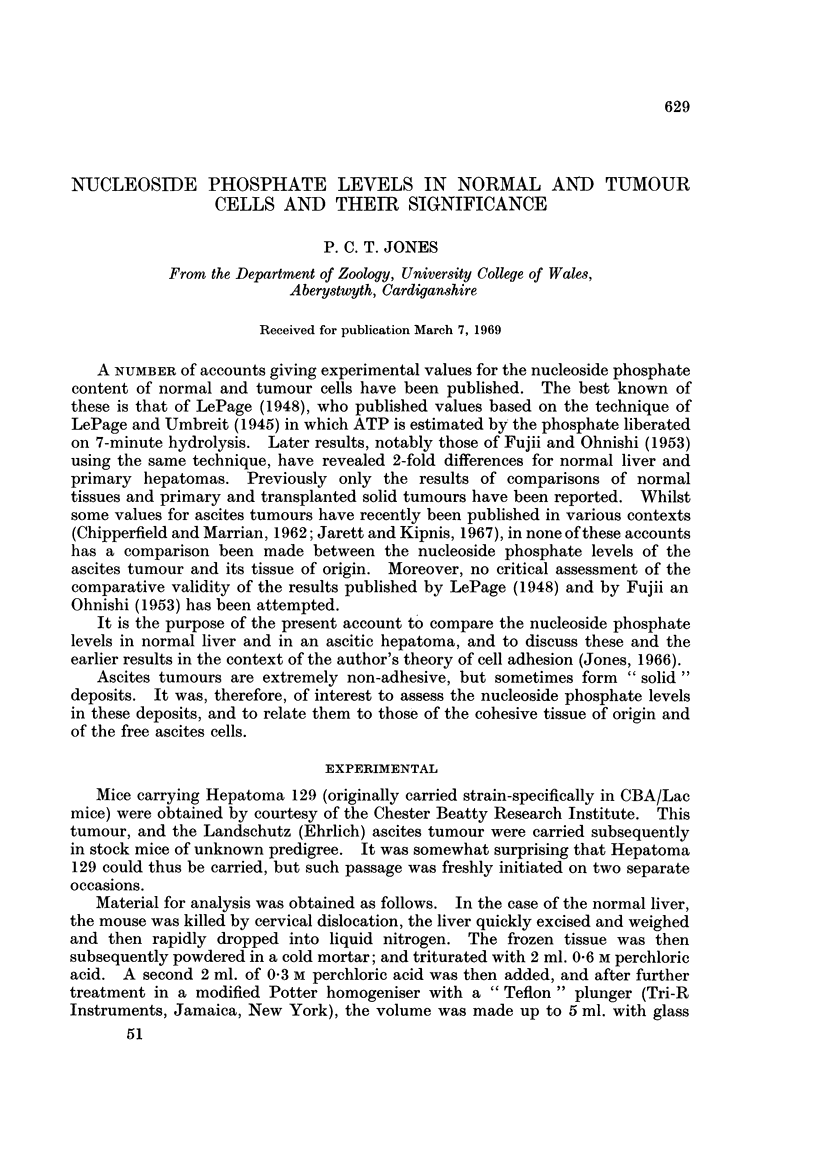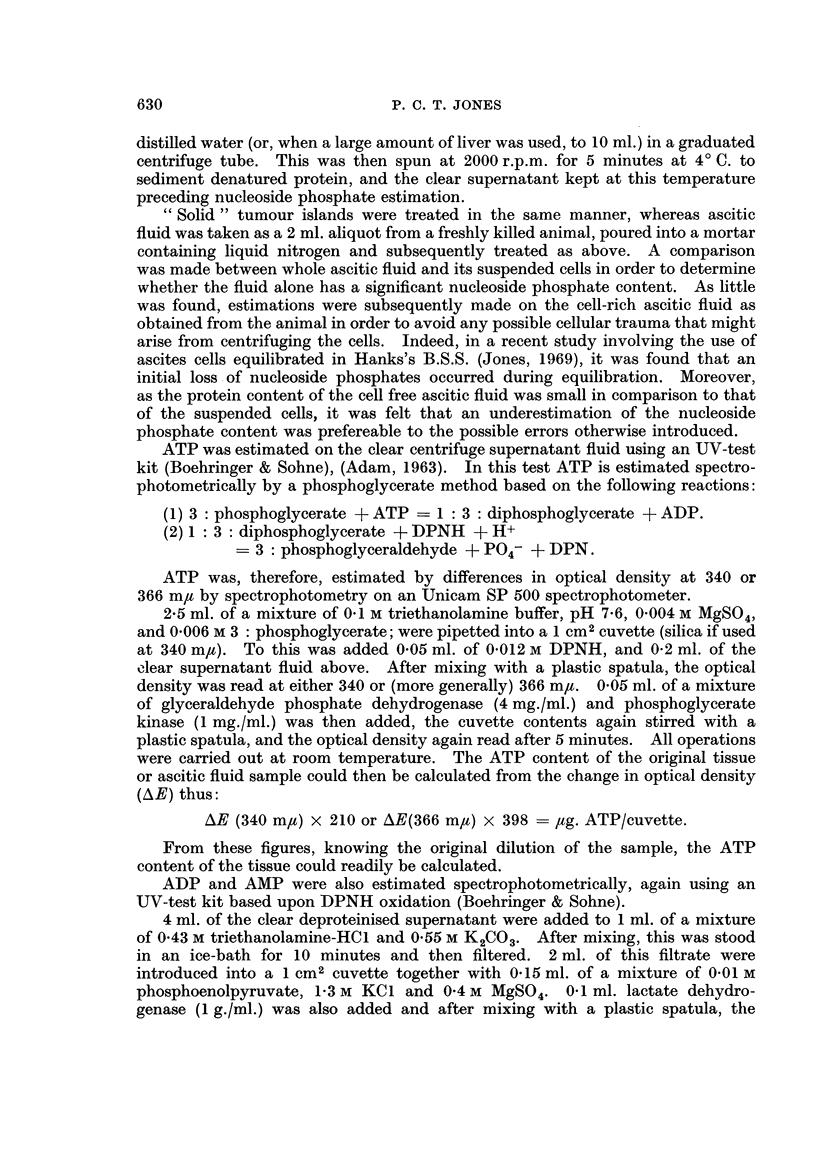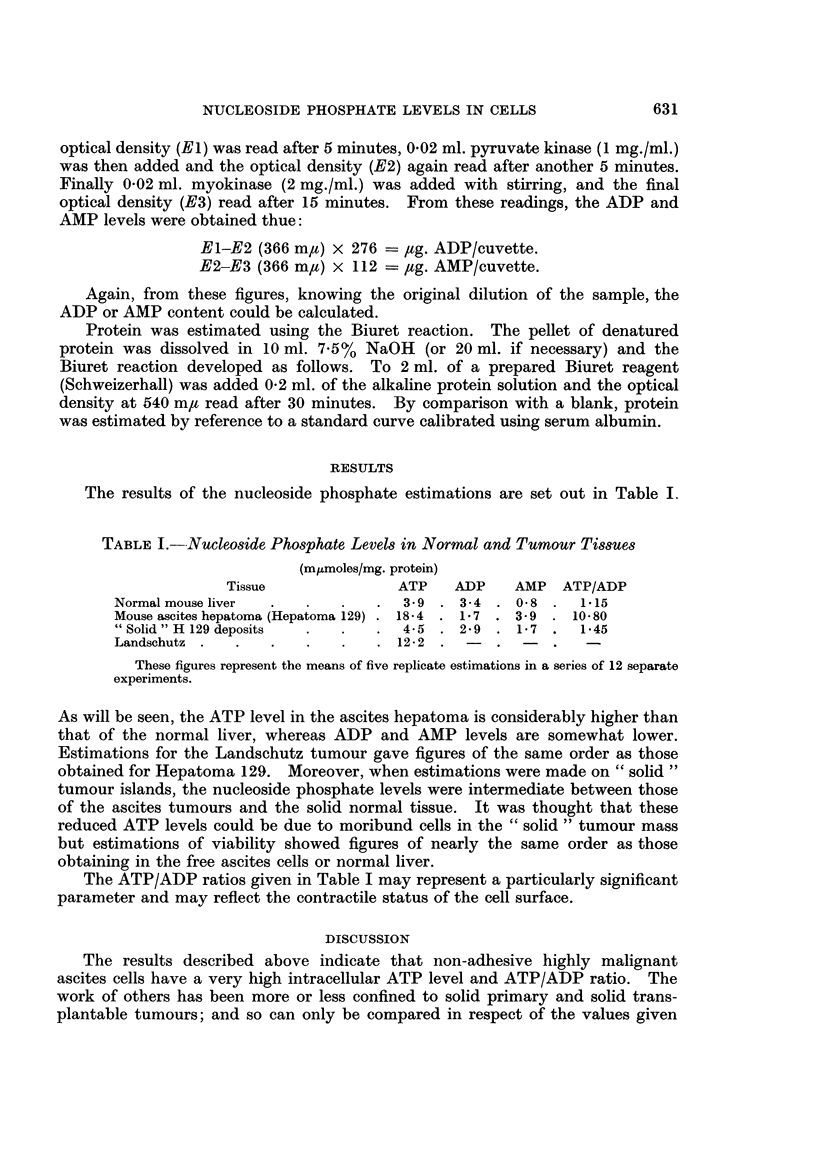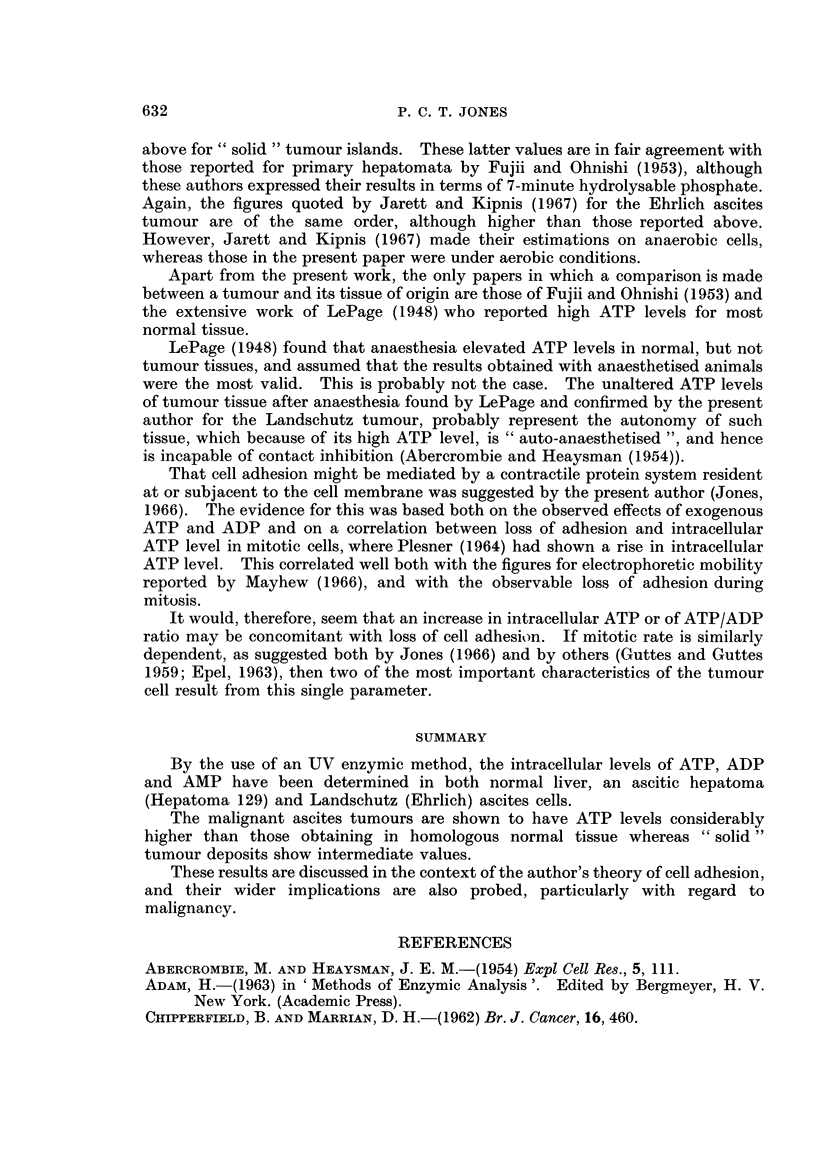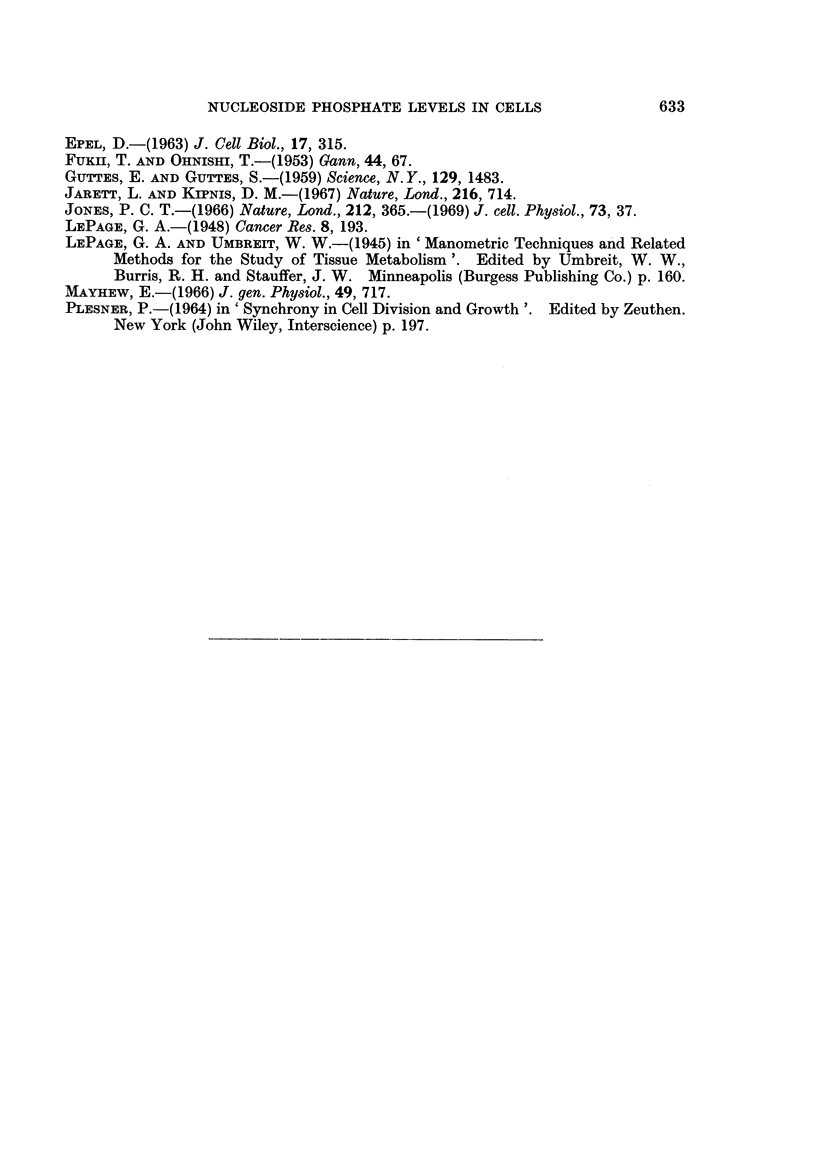# Nucleoside phosphate levels in normal and tumour cells and their significance.

**DOI:** 10.1038/bjc.1969.77

**Published:** 1969-09

**Authors:** P. C. Jones


### NUCLEOSIDE PHOSPHATE LEVELS IN NORMAL AND TUMOUR CELLS AND THEIR SIGNIFICANCE

P. C. T. JONES

*From the Department of Zoology, University College of Wales, Aberystwyth, Cardiganshire*

Received for publication March 7, 1969

A NUMBER of accounts giving experimental values for the nucleoside phosphate content of normal and tumour cells have been published. The best known of these is that of LePage (1948), who published values based on the technique of LePage and Umbreit (1945) in which ATP is estimated by the phosphate liberated on 7-minute hydrolysis. Later results, notably those of Fujii and Ohnishi (1953) using the same technique, have revealed 2-fold differences for normal liver and primary hepatomas. Previously only the results of comparisons of normal tissues and primary and transplanted solid tumours have been reported. Whilst some values for ascites tumours have recently been published in various contexts (Chipperfield and Marrian, 1962; Jarett and Kipnis, 1967), in none of these accounts has a comparison been made between the nucleoside phosphate levels of the ascites tumour and its tissue of origin. Moreover, no critical assessment of the comparative validity of the results published by LePage (1948) and by Fujii an Ohnishi (1953) has been attempted.

It is the purpose of the present account to compare the nucleoside phosphate levels in normal liver and in an ascitic hepatoma, and to discuss these and the earlier results in the context of the author's theory of cell adhesion (Jones, 1966).

Ascites tumours are extremely non-adhesive, but sometimes form "solid" deposits. It was, therefore, of interest to assess the nucleoside phosphate levels in these deposits, and to relate them to those of the cohesive tissue of origin and of the free ascites cells.

### EXPERIMENTAL

Mice carrying Hepatoma 129 (originally carried strain-specifically in CBA/Lac mice) were obtained by courtesy of the Chester Beatty Research Institute. This tumour, and the Landschutz (Ehrlich) ascites tumour were carried subsequently in stock mice of unknown predigree. It was somewhat surprising that Hepatoma 129 could thus be carried, but such passage was freshly initiated on two separate occasions.

Material for analysis was obtained as follows. In the case of the normal liver, the mouse was killed by cervical dislocation, the liver quickly excised and weighed and then rapidly dropped into liquid nitrogen. The frozen tissue was then subsequently powdered in a cold mortar; and triturated with 2 ml. 0·6 M perchloric acid. A second 2 ml. of 0·3 M perchloric acid was then added, and after further treatment in a modified Potter homogeniser with a "Teflon" plunger (Tri-R Instruments, Jamaica, New York), the volume was made up to 5 ml. with glass distilled water (or, when a large amount of liver was used, to 10 ml.) in a graduated centrifuge tube. This was then spun at 2000 r.p.m. for 5 minutes at 4° C. to sediment denatured protein, and the clear supernatant kept at this temperature preceding nucleoside phosphate estimation.

" Solid " tumour islands were treated in the same manner, whereas ascitic fluid was taken as a 2 ml. aliquot from a freshly killed animal, poured into a mortar containing liquid nitrogen and subsequently treated as above. A comparison was made between whole ascitic fluid and its suspended cells in order to determine whether the fluid alone has a significant nucleoside phosphate content. As little was found, estimations were subsequently made on the cell-rich ascitic fluid as obtained from the animal in order to avoid any possible cellular trauma that might arise from centrifuging the cells. Indeed, in a recent study involving the use of ascites cells equilibrated in Hanks's B.S.S. (Jones, 1969), it was found that an initial loss of nucleoside phosphates occurred during equilibration. Moreover, as the protein content of the cell free ascitic fluid was small in comparison to that of the suspended cells, it was felt that an underestimation of the nucleoside phosphate content was prefereable to the possible errors otherwise introduced.

ATP was estimated on the clear centrifuge supernatant fluid using an UV-test kit (Boehringer & Sohne), (Adam, 1963). In this test ATP is estimated spectrophotometrically by a phosphoglycerate method based on the following reactions:

(1) 3 : phosphoglycerate + ATP = 1 : 3 : diphosphoglycerate + ADP.
(2) 1 : 3 : diphosphoglycerate + DPNH + $H^+$
= 3 : phosphoglyceraldehyde + $PO_4^-$ + DPN.

ATP was, therefore, estimated by differences in optical density at 340 or 366 m$\mu$ by spectrophotometry on an Unicam SP 500 spectrophotometer.

2·5 ml. of a mixture of 0·1 M triethanolamine buffer, pH 7·6, 0·004 M $MgSO_4$, and 0·006 M 3 : phosphoglycerate; were pipetted into a 1 $cm^2$ cuvette (silica if used at 340 m$\mu$). To this was added 0·05 ml. of 0·012 M DPNH, and 0·2 ml. of the clear supernatant fluid above. After mixing with a plastic spatula, the optical density was read at either 340 or (more generally) 366 m$\mu$. 0·05 ml. of a mixture of glyceraldehyde phosphate dehydrogenase (4 mg./ml.) and phosphoglycerate kinase (1 mg./ml.) was then added, the cuvette contents again stirred with a plastic spatula, and the optical density again read after 5 minutes. All operations were carried out at room temperature. The ATP content of the original tissue or ascitic fluid sample could then be calculated from the change in optical density ($\Delta E$) thus:

$$\Delta E \text{ (340 m}\mu) \times 210 \text{ or } \Delta E\text{(366 m}\mu) \times 398 = \mu\text{g. ATP/cuvette.}$$

From these figures, knowing the original dilution of the sample, the ATP content of the tissue could readily be calculated.

ADP and AMP were also estimated spectrophotometrically, again using an UV-test kit based upon DPNH oxidation (Boehringer & Sohne).

4 ml. of the clear deproteinised supernatant were added to 1 ml. of a mixture of 0·43 M triethanolamine-HCl and 0·55 M $K_2CO_3$. After mixing, this was stood in an ice-bath for 10 minutes and then filtered. 2 ml. of this filtrate were introduced into a 1 $cm^2$ cuvette together with 0·15 ml. of a mixture of 0·01 M phosphoenolpyruvate, 1·3 M KCl and 0·4 M $MgSO_4$. 0·1 ml. lactate dehydrogenase (1 g./ml.) was also added and after mixing with a plastic spatula, the optical density ($E1$) was read after 5 minutes, 0·02 ml. pyruvate kinase (1 mg./ml.) was then added and the optical density ($E2$) again read after another 5 minutes. Finally 0·02 ml. myokinase (2 mg./ml.) was added with stirring, and the final optical density ($E3$) read after 15 minutes. From these readings, the ADP and AMP levels were obtained thue:

$$E1\text{–}E2\ (366\ m\mu) \times 276 = \mu g.\ \text{ADP/cuvette.}$$

$$E2\text{–}E3\ (366\ m\mu) \times 112 = \mu g.\ \text{AMP/cuvette.}$$

Again, from these figures, knowing the original dilution of the sample, the ADP or AMP content could be calculated.

Protein was estimated using the Biuret reaction. The pellet of denatured protein was dissolved in 10 ml. 7·5% NaOH (or 20 ml. if necessary) and the Biuret reaction developed as follows. To 2 ml. of a prepared Biuret reagent (Schweizerhall) was added 0·2 ml. of the alkaline protein solution and the optical density at 540 mμ read after 30 minutes. By comparison with a blank, protein was estimated by reference to a standard curve calibrated using serum albumin.

### RESULTS

The results of the nucleoside phosphate estimations are set out in Table I.

TABLE I.—*Nucleoside Phosphate Levels in Normal and Tumour Tissues*

(mμmoles/mg. protein)

| Tissue | ATP | ADP | AMP | ATP/ADP |
|---|---|---|---|---|
| Normal mouse liver | 3·9 | 3·4 | 0·8 | 1·15 |
| Mouse ascites hepatoma (Hepatoma 129) | 18·4 | 1·7 | 3·9 | 10·80 |
| " Solid " H 129 deposits | 4·5 | 2·9 | 1·7 | 1·45 |
| Landschutz | 12·2 | — | — | — |

These figures represent the means of five replicate estimations in a series of 12 separate experiments.

As will be seen, the ATP level in the ascites hepatoma is considerably higher than that of the normal liver, whereas ADP and AMP levels are somewhat lower. Estimations for the Landschutz tumour gave figures of the same order as those obtained for Hepatoma 129. Moreover, when estimations were made on " solid " tumour islands, the nucleoside phosphate levels were intermediate between those of the ascites tumours and the solid normal tissue. It was thought that these reduced ATP levels could be due to moribund cells in the " solid " tumour mass but estimations of viability showed figures of nearly the same order as those obtaining in the free ascites cells or normal liver.

The ATP/ADP ratios given in Table I may represent a particularly significant parameter and may reflect the contractile status of the cell surface.

### DISCUSSION

The results described above indicate that non-adhesive highly malignant ascites cells have a very high intracellular ATP level and ATP/ADP ratio. The work of others has been more or less confined to solid primary and solid transplantable tumours; and so can only be compared in respect of the values given above for " solid " tumour islands. These latter values are in fair agreement with those reported for primary hepatomata by Fujii and Ohnishi (1953), although these authors expressed their results in terms of 7-minute hydrolysable phosphate. Again, the figures quoted by Jarett and Kipnis (1967) for the Ehrlich ascites tumour are of the same order, although higher than those reported above. However, Jarett and Kipnis (1967) made their estimations on anaerobic cells, whereas those in the present paper were under aerobic conditions.

Apart from the present work, the only papers in which a comparison is made between a tumour and its tissue of origin are those of Fujii and Ohnishi (1953) and the extensive work of LePage (1948) who reported high ATP levels for most normal tissue.

LePage (1948) found that anaesthesia elevated ATP levels in normal, but not tumour tissues, and assumed that the results obtained with anaesthetised animals were the most valid. This is probably not the case. The unaltered ATP levels of tumour tissue after anaesthesia found by LePage and confirmed by the present author for the Landschutz tumour, probably represent the autonomy of such tissue, which because of its high ATP level, is " auto-anaesthetised ", and hence is incapable of contact inhibition (Abercrombie and Heaysman (1954)).

That cell adhesion might be mediated by a contractile protein system resident at or subjacent to the cell membrane was suggested by the present author (Jones, 1966). The evidence for this was based both on the observed effects of exogenous ATP and ADP and on a correlation between loss of adhesion and intracellular ATP level in mitotic cells, where Plesner (1964) had shown a rise in intracellular ATP level. This correlated well both with the figures for electrophoretic mobility reported by Mayhew (1966), and with the observable loss of adhesion during mitosis.

It would, therefore, seem that an increase in intracellular ATP or of ATP/ADP ratio may be concomitant with loss of cell adhesion. If mitotic rate is similarly dependent, as suggested both by Jones (1966) and by others (Guttes and Guttes 1959; Epel, 1963), then two of the most important characteristics of the tumour cell result from this single parameter.

### SUMMARY

By the use of an UV enzymic method, the intracellular levels of ATP, ADP and AMP have been determined in both normal liver, an ascitic hepatoma (Hepatoma 129) and Landschutz (Ehrlich) ascites cells.

The malignant ascites tumours are shown to have ATP levels considerably higher than those obtaining in homologous normal tissue whereas " solid " tumour deposits show intermediate values.

These results are discussed in the context of the author's theory of cell adhesion, and their wider implications are also probed, particularly with regard to malignancy.

### REFERENCES

ABERCROMBIE, M. AND HEAYSMAN, J. E. M.—(1954) *Expl Cell Res.*, **5**, 111.

ADAM, H.—(1963) in ' Methods of Enzymic Analysis '. Edited by Bergmeyer, H. V. New York. (Academic Press).

CHIPPERFIELD, B. AND MARRIAN, D. H.—(1962) *Br. J. Cancer*, **16**, 460.

EPEL, D.—(1963) *J. Cell Biol.*, **17**, 315.
FUKII, T. AND OHNISHI, T.—(1953) *Gann*, **44**, 67.
GUTTES, E. AND GUTTES, S.—(1959) *Science, N.Y.*, **129**, 1483.
JARETT, L. AND KIPNIS, D. M.—(1967) *Nature, Lond.*, **216**, 714.
JONES, P. C. T.—(1966) *Nature, Lond.*, **212**, 365.—(1969) *J. cell. Physiol.*, **73**, 37.
LEPAGE, G. A.—(1948) *Cancer Res.* **8**, 193.
LEPAGE, G. A. AND UMBREIT, W. W.—(1945) in ' Manometric Techniques and Related Methods for the Study of Tissue Metabolism '. Edited by Umbreit, W. W., Burris, R. H. and Stauffer, J. W. Minneapolis (Burgess Publishing Co.) p. 160.
MAYHEW, E.—(1966) *J. gen. Physiol.*, **49**, 717.
PLESNER, P.—(1964) in ' Synchrony in Cell Division and Growth '. Edited by Zeuthen. New York (John Wiley, Interscience) p. 197.